# The history of introduction of the African baobab (*Adansonia digitata*, Malvaceae: Bombacoideae) in the Indian subcontinent

**DOI:** 10.1098/rsos.150370

**Published:** 2015-09-09

**Authors:** Karen L. Bell, Haripriya Rangan, Christian A. Kull, Daniel J. Murphy

**Affiliations:** 1 Royal Botanic Gardens Victoria, Melbourne, Victoria 3004, Australia; 2 School of Geography, University of Melbourne, Victoria 3053, Australia; 3 Institut de Géographie et Durabilité, Université de Lausanne, Lausanne 1015, Switzerland

**Keywords:** *Adansonia digitata*, genetic diversity, Indian subcontinent, population structure, introduction pathways, population genetics

## Abstract

To investigate the pathways of introduction of the African baobab, *Adansonia digitata*, to the Indian subcontinent, we examined 10 microsatellite loci in individuals from Africa, India, the Mascarenes and Malaysia, and matched this with historical evidence of human interactions between source and destination regions. Genetic analysis showed broad congruence of African clusters with biogeographic regions except along the Zambezi (Mozambique) and Kilwa (Tanzania), where populations included a mixture of individuals assigned to at least two different clusters. Individuals from West Africa, the Mascarenes, southeast India and Malaysia shared a cluster. Baobabs from western and central India clustered separately from Africa. Genetic diversity was lower in populations from the Indian subcontinent than in African populations, but the former contained private alleles. Phylogenetic analysis showed Indian populations were closest to those from the Mombasa-Dar es Salaam coast. The genetic results provide evidence of multiple introductions of African baobabs to the Indian subcontinent over a longer time period than previously assumed. Individuals belonging to different genetic clusters in Zambezi and Kilwa may reflect the history of trafficking captives from inland areas to supply the slave trade between the fifteenth and nineteenth centuries. Baobabs in the Mascarenes, southeast India and Malaysia indicate introduction from West Africa through eighteenth and nineteenth century European colonial networks.

## 1. Background

Innumerable plant species have been transported by humans around the world for a variety of practical and cultural uses. Many of these have geographically widespread or disjunct distributions, with no historical records for when and how they may have been dispersed from their places of origin to new sites [1]. Several studies of cultivated crops (e.g. [2–5]) and weeds (e.g. [6–8]) have sought to investigate the origins of taxa that lack historical documentation by using population genetics to match genotypes in the introduced and native ranges in order to determine source populations. However, genetic evidence of source populations is often insufficient for inferring agency, modes and timing of plant dispersals from their places of origin to new locations. Other evidence, such as archaeobotanical remains, can provide an indication of when the plants may have arrived and indicate possible agents and pathways of introduction [5]. Additional cultural evidence can come from comparing plant terms and uses between places of origin and introduction [9,10]. Combining all these forms of evidence can provide a more comprehensive historical understanding of the global biogeography of plant dispersals and distributions.

The charismatic tree genus *Adansonia* (Malvaceae: Bombacoideae) occurs in Africa, Madagascar and Australia, with a different lineage present in each land mass [11–13]. Molecular divergence dating suggests that the three main lineages shared a common ancestor 5–15 Ma (during the Miocene), with the current distribution hypothesized to be the result of long-distance hydrochory [12,14]. *Adansonia digitata* L., or the African baobab, is widely distributed across continental Africa. *Adansonia digitata s.l.* (i.e. in the broad sense) encompasses two described species, the tetraploid *A. digitata* *s*.*s*. and the diploid *A. kilima* [12]. The distribution of *A. digitata s.s.* extends across much of Sub-Saharan Africa, from the northern Sahel to South Africa's Limpopo province, but does not occur in the rainforest areas of central Africa, or higher altitude localities (above 800 m elevation) of eastern Africa [13]. Phylogeographic analyses have demonstrated populations of *A. digitata s.l.* in eastern and western Africa to be genetically distinct, with the eastern populations monophyletic within a paraphyletic grade of western populations [15]. *Adansonia kilima* is recorded in higher altitude localities (650–1500 m elevation), partially overlapping with the range of *A. digitata s.s.*, from the eastern slopes of Kilimanjaro to northern South Africa, and west to the Caprivi Strip, Namibia [12]. Phylogenetic analysis has demonstrated that *A. digitata* and *A. kilima* are genetically similar, suggesting that tetraploidy evolved relatively recently [12]. In this study, we refer to this species-complex as *A. digitata* and hereafter use its common name, baobab.

Biogeographic studies of baobabs in the African continent show that there are close associations between past human settlements and the presence of the species [13,16]. The baobab is highly valued for a variety of food and artisanal uses and for its cultural symbolism extending over millennia [13,17–22]. The fruit has a powdery pulp surrounding numerous seeds with a hard endocarp. The sweet–sour pulp is particularly favoured and widely consumed, but the seeds are difficult to digest without additional processing. In some parts of Africa, the seeds are eaten after roasting or grinding into flour [23]. Most commonly though, the seed is discarded or passed intact through the digestive tract after eating the pulp. This may be the means by which human-mediated dispersal of the baobab occurred. Genetic studies also suggest that humans have been the prime agents responsible for distributing the tree species across the African continent [15].

The role of human agency in baobab dispersal is evident in its presence in the Caribbean and parts of tropical South America, where enslaved people were transported from West Africa between the sixteenth and nineteenth centuries to work on sugarcane (*Saccharum* spp.) plantations [24]. African baobab populations are also found in the Indian subcontinent and Sri Lanka [25–27], with sporadic distribution in various places around the Indian Ocean including Yemen, southern Iran [28], Comoros, northwest Madagascar and the Mascarene Islands [25,29], Malaysia and Indonesia [30]. The history of dispersal to these places is less well known. Although it has not been previously hypothesized, long-distance hydrochory is a possibility. Baum *et al.* [14] provided evidence that *Adansonia* in northwest Australia arrived from Africa via long-distance oceanic dispersal in the Miocene. The fruit of the African baobab has been demonstrated to remain viable after immersion in seawater for six months [15]. This could explain the coastal presence of the African baobab on the Indian subcontinent and other locations around the Indian Ocean. However, the lack of species-level divergence, according to morphological taxonomy, between baobabs in Africa and those found in the Indian subcontinent [11,13] would suggest a recent dispersal. This makes human-assisted dispersal a more plausible alternative than transoceanic drift.

The few published surveys of baobabs in the Indian subcontinent attribute the introduction of baobabs to Arab traders [26,27] or medieval Muslim rulers in the subcontinent that maintained African slave armies [13,25], but without any supporting evidence from genetic, archaeobotanical or ethno-historical analyses [31]. These hypotheses imply a recent history of the baobab in the Indian subcontinent which stand in contrast to significant evidence of a deep prehistory of biotic exchange between Africa and the Indian subcontinent [32–37]. These exchanges include the introduction of cereals such as sorghum (*Sorghum bicolor*), pearl millet (*Pennisetum glaucum*) and finger millet (*Eleusine coracana*), and legumes, such as cowpea (*Vigna unguiculata*), and hyacinth bean (*Lablab purpureus*) from Africa into the Indian subcontinent during the second and first millennia BCE [38–42], and the movement of zebu cattle (*Bos indicus*) in the reverse direction [43]. There was also possibly an earlier introduction of tamarind (*Tamarindus indica*) from the Sudano-Sahelian region to the Indian subcontinent [44–46]. We hypothesize that the baobab, with similarly important nutritious and other use values, may have also been introduced from Africa to the Indian subcontinent in prehistoric times.

In this study, we investigate the history and pathways of introduction of baobabs from Africa to the Indian subcontinent and other parts of the Indian Ocean region. We combined genetic analysis of inferred ancestry and phylogeography with historical evidence of oceanic trade and migrations across the Indian Ocean to: (i) determine source populations of baobabs by examining genetic variation and relationships within and between African, Indian subcontinent and other Indian Ocean populations, (ii) associate these source populations to specific dispersal pathways based on historical trade routes and networks, and (iii) infer possible time periods of introductions based on genetic and historical evidence.

## 2. Material and methods

### 2.1 Sampling

Our sampling in Africa focused on the eastern lineage, based on the assumption that this was the most likely source for dispersal of baobabs across the Indian Ocean. Thorough surveys of baobabs were conducted in Mozambique and Tanzania, with further sampling in Kenya and South Africa. A single sample was included from Senegal as a reference sample for baobabs in the West African genetic group [15]. Baobabs were sampled from multiple introduced populations in India, along with three individuals from the islands of Réunion and Mauritius, and one individual from Penang, in Malaysia. For details, see the electronic supplementary material, appendices S1 and S2.

Leaves were collected into plastic bags and dried with silica gel. If trees were not in leaf, then bark samples were used. Although bark samples are more likely than leaf samples to contain DNA from other organisms, such as endophytes and insects, it is highly unlikely that the *A. digitata*-specific primers would amplify them. To confirm that this had not occurred, we checked results from these samples for the presence of more than four bands per locus, to confirm that no extraneous DNA had amplified.

Since baobab trees tend to occur as isolated individuals throughout their range rather than forming distinct populations, in many locations one tree was sampled. However, for some populations, samples were taken from up to 19 individuals. Voucher specimens were retained for a representative subset of individuals and deposited in the National Herbarium of Victoria (MEL).

### 2.2 DNA isolation and microsatellite genotyping

Plant DNA was isolated from samples using DNeasy Plant Mini Kits (QIAGEN), with modifications to the manufacturer's protocol as described by Pettigrew *et al.* [12]. The microsatellite loci Ad01, Ad02, Ad06, Ad08, Ad09, Ad12, Ad13, Ad14, Ad15 and Ad18 [47] were amplified following the method of James *et al.* [48], modified for multiplexing polymerase chain reactions (PCRs) using the Type-It Microsatellite PCR Kit (QIAGEN). Amplification reactions contained a final concentration of 1x PCR Master Mix (QIAGEN), 0.075 μM each multiplexed forward primer appended to the 454A sequencing tag (Applied Biosystems, Foster City, CA, USA), 0.25 μM each reverse primer, 0.1 μM per multiplexed locus of 454A sequencing tag labelled with either 6-FAM, NED, HEX or PET (Applied Biosystems). Thermal cycling followed the instructions provided with the Type-It Kit. Specifically, initial heat activation of 5 min at 95°C was followed by 28 cycles of denaturation for 30 s at 95°C, annealing for 90 s at 60°C, and extension for 30 s at 72°C, with a final extension of 30 min at 60°C. Following PCR, amplifications using compatible dye types were diluted to equal concentrations and combined, then separated on an ABI 3730XL sequencer with a GS500LIZ size standard (Applied Biosystems) at Macrogen Inc. (Seoul, Korea). Allele sizes were scored using the Geneious microsatellite plugin v. 1.0.0 (Biomatters Ltd).

### 2.3 Genetic diversity

Allele frequencies and measures of genetic diversity were examined for three sets of data: the entire range of the baobab, African populations only and Indian populations only. This allowed us to assess whether any changes could be detected in response to founder effects or admixture of lineages, which may be associated with recent introduction to India, and to test for *in situ* evolution, which may have occurred under the scenario of an ancient introduction. Specifically, we used GenoDive to test whether populations and loci were in Hardy–Weinberg equilibrium (HWE), and we used SPAGeDi v. 1.3a [49] to calculate the number of alleles per locus, expected heterozygosity under HWE, inbreeding coefficient (*F*_is_) and number of private alleles.

To examine whether there was a recent reduction in effective population size of baobabs, as would be expected from a founder effect, deviations from mutation-drift equilibrium were tested using Bottleneck v. 1.2.02 [50]. Expected heterozygosities were simulated with 1000 replications under the Two Phased Model of Mutation with default settings (variance 30, 70% of mutations fitting the stepwise mutation model). Three statistical tests are available in the Bottleneck software to compare observed versus expected heterozygosities. The ‘standardized differences test’ is unsuitable for less than 20 loci [50]. We therefore used the ‘sign test’ and ‘Wilcoxon sign-rank test’, which are less sensitive to the low number of loci, and the allele frequency distribution was compared to that expected under mutation-drift equilibrium.

### 2.4 Population structure

A Bayesian classification scheme was used to identify genetic clusters and evaluate their spatial distribution, in order to determine potential connections between source and introduced populations and to assess overall genetic structure. This analysis was implemented in Structure v. 2.3.3 [51], without *a priori* grouping of individuals into populations. In tetraploid species such as the baobab, the genotype of partial heterozygotes (with two to three different alleles at a locus) is ambiguous. To avoid making assumptions about the genotypes of these individuals, we created a binary matrix of presence or absence of allele variants and treated these as dominant markers [52]. An admixture model, with loci assumed to be unlinked, was used to calculate the likelihood of different values for the number of clusters (*K*). Simulations consisted of a burn-in of 50 000 iterations followed by 100 000 Markov chain Monte Carlo iterations. Ten runs for each *K*=1–15 were carried out on an SGI Altix XE Cluster through the Victorian Life Sciences Computing Initiative. The value(s) of *K* that best explained the structure in the data was/were determined using Structure Harvester web v. 0.6.93 [53], following the Δ*K* method of Evanno *et al.* [54]. To assign individuals to genetic clusters, multiple runs at the selected value of *K* were combined using Clumpp v. 1.1.2 [55], and plotted graphically using Distruct v. 1.1 [56]. Cluster assignment of individuals and populations was visualized spatially using PhyloGeoViz [57].

The statistical significance of spatial genetic clustering identified in the Structure analysis, for selected values of *K*, was tested using analysis of molecular variance (AMOVA) [58] performed in Arlecore v. 3.5.1.3 [59]. Populations were allocated to regions, based on the predominant Structure cluster to which the individuals of that population belonged. Samples from West Africa, the Mascarene Islands and Malaysia were excluded due to insufficient numbers. Analyses were based on the *F*_ST_ measure of genetic diversity, with 9999 permutations.

Isolation by distance (IBD) was examined among individuals through a Mantel test (linearized genetic distance versus ln (1+geographical distance, to ensure that this number is non-zero); 999 permutations) conducted in Genalex v. 6.41 [60]. Genetic distance was calculated as the binary genetic distance in Genalex. Since geographical structure in the form of genetic clustering and in the form of IBD can be confounded, further Mantel tests and partial Mantel tests were carried out to limit this effect using the vegan package [61] in R [62]. A Mantel test of genetic distance versus geographical distance, with 999 permutations, was carried out within the geographical regions (defined above) to remove the confounding effect of genetic clustering from the test of IBD within a region. Conversely, a partial Mantel test of genetic distance versus region (0 for samples from the same region, 1 for samples from different regions), with geographical distance as a covariate, was used to test whether there is genetic structure that cannot be solely explained as IBD [63].

A principal coordinates analysis (PCoA), conducted using Genalex v. 6.41 [60], was also used to examine genetic clustering defined by Structure at *K*=7. Clusters with disjunct distribution were subdivided into multiple populations and samples with missing data were excluded from the analysis. This left a total of seven groups under consideration. Principal coordinates were calculated based on the simple genetic distance (i.e. the total number of allelic differences) between each pair of samples, via a covariance matrix with data standardization. The first two principal coordinates were retained and plotted.

Intraspecific phylogenetic analysis was used to investigate the relationships between regions and to provide further evidence for inferring source populations and dispersal pathways. The biogeographic regions as defined by Structure at *K*=7 were used, with the inclusion of West Africa and the Mascarenes, and individuals were assigned to these regions. Loci were bootstrapped to generate 1000 pseudoreplicates and genetic distances between regions were calculated with the *D*_C_ method [64] for each bootstrapped data matrix in Phylip v. 3.69 [65]. These distances were then used to construct unrooted neighbour-joining trees, and a 50% majority-rule bootstrap consensus tree was generated in Phylip.

## 3. Results

### 3.1 Genetic diversity

In general, genetic diversity parameters were found to be lower in baobabs from India compared to Africa. The number of alleles per locus and heterozygosity were both lower in India, although *F*_is_ values were similar and a small number of private alleles were present in the Indian samples (table 1). Measures of G_IS_ within populations and loci frequently demonstrated deviation from HWE (electronic supplementary material, appendix S3). Tests for deviations from mutation-drift equilibrium, based on the ‘sign test’ and ‘Wilcoxon test’ in Bottleneck, were non-significant under all statistical tests, and allele frequencies showed a normal L-shaped distribution. This implies that there have been no recent bottlenecks or reductions in effective population size [50]. However, since tests for genetic bottlenecks based on deviations from mutation-drift equilibrium have low sensitivity [50,66], we cannot rule out the possibility of much older bottlenecks for baobabs in the Indian subcontinent.

**Table 1.** Population statistics (mean±s.d. across loci) for all *Adansonia digitata* populations, for African populations only and for Indian populations only. *N*_A_, number of alleles per locus; *H*_e_, expected heterozygosity; *F*_is_, inbreeding coefficient.

region	*n*	*N*_A_	private alleles	*H*_e_	*F*_is_
Africa	106	15.5±3.6	5.8±1.9	0.823±0.113	−0.011±0.108
India	35	10.2±3.5	0.9±1.0	0.773±0.153	−0.006±0.098
India (excluding Chennai)	34	10.2±3.5	0.6±0.8	0.766±0.160	−0.008±0.101
all	144	16.2±3.7	n.a.	0.819±0.119	−0.007±0.095

### 3.2 Population structure

**Figure 1.**
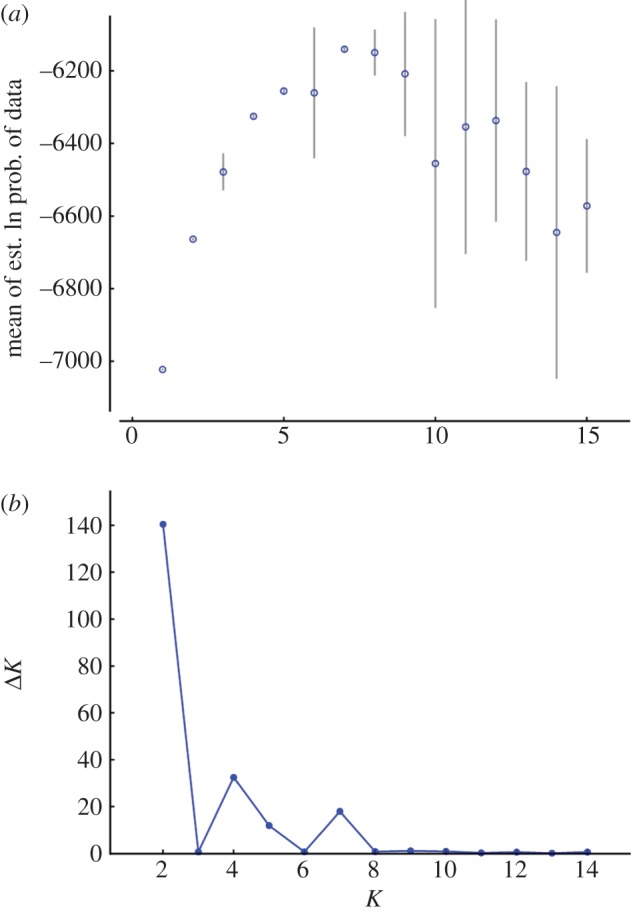
Comparison of the results of Structure analyses for genetic clustering of *Adansonia digitata* for *K* (number of clusters) between 1 and 15. (*a*) Mean and standard deviation of ln(likelihood) for each value of *K*. (*b*) Δ*K* statistic of Evanno *et al.* [54] for each value of *K*.

**Figure 2.**
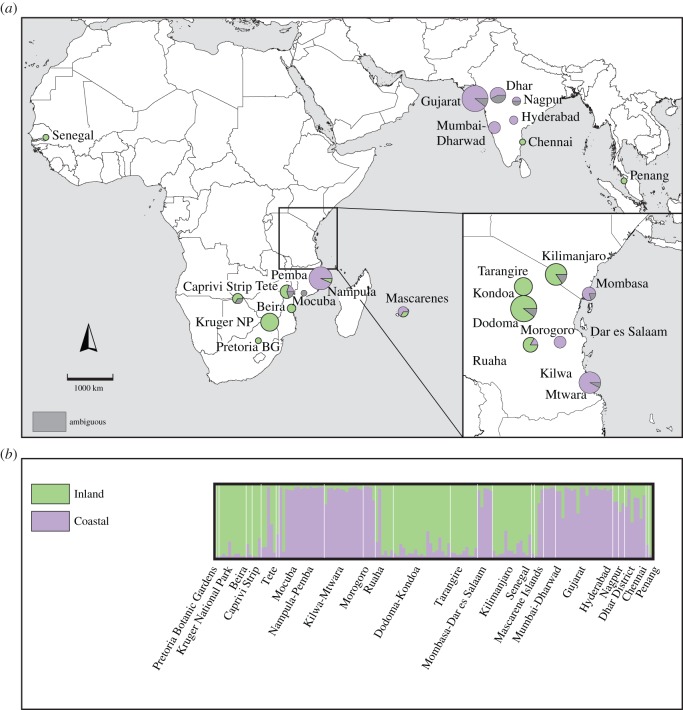
Structure analysis at *K*=2. (*a*) Geographical localities of *Adansonia digitata* populations, and proportion of individuals within populations belonging to each genetic cluster. Assignment is considered to be ambiguous if the *Q*-value is less than 0.75. The size of the pie chart is proportional to the number of sampled individuals. (*b*) Proportional assignment of *Adansonia digitata* individuals to genetic clusters.

**Figure 3.**
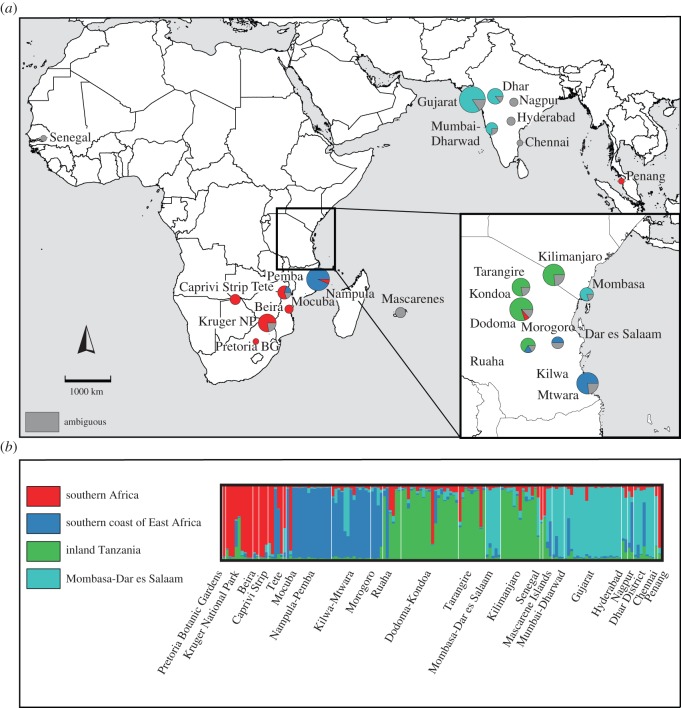
Structure analysis at *K*=4. (*a*) Geographical localities of *Adansonia digitata* populations, and proportion of individuals within populations belonging to each genetic cluster. Assignment is considered to be ambiguous if the *Q*-value is less than 0.75. The size of the pie chart is proportional to the number of sampled individuals. (*b*) Proportional assignment of *Adansonia digitata* individuals to genetic clusters.

**Figure 4.**
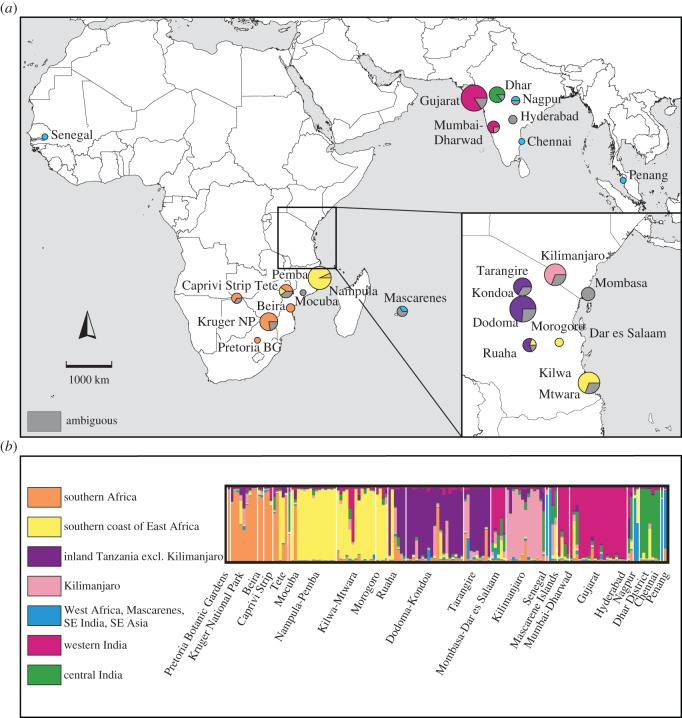
Structure analysis at *K*=7. (*a*) Geographical localities of *Adansonia digitata* populations, and proportion of individuals within populations belonging to each genetic cluster. Assignment is considered to be ambiguous if the *Q*-value is less than 0.75. The size of the pie chart is proportional to the number of sampled individuals. (*b*) Proportional assignment of *Adansonia digitata* individuals to genetic clusters.

Likelihood values for Structure runs at different values of *K* increased rapidly with increasing *K*, up to *K*=4, after which the likelihood increased more slowly or decreased, and the standard deviations were greater (figure 1*a*). The highest value for Δ*K*, indicating the number of clusters that best explained the data [54], was recorded at *K*=2 (figure 1*b*). A peak at *K*=2 in Structure analyses can be an artefact (e.g. [42,67]), so we also examined clustering based on the secondary peaks at *K*=4 and 7. The inferred clusters at higher *K*-values nested within the clusters inferred at lower *K*-values (figures 2–4). Most individuals were assigned to populations with high certainty at each of these values of *K*, although a small number of individuals were ambiguously assigned. Across all *K*-values, *α* was low, implying that these ambiguous assignments are due to uncertainty of origin, rather than admixture.

At *K*=2, the samples were divided into an Inland and a Coastal cluster (figure 2*a*,*b*). The Inland cluster included individuals from inland East Africa, southeast India (Chennai), Malaysia and Senegal. The Coastal cluster included individuals from coastal and lowland East Africa and India (excluding Chennai). Individuals from Tete, in Mozambique's Zambezi River valley, and from Réunion and Mauritius included a mixture of both clusters.

At *K*=4, the clusters were: (1) southern Africa (SA) and Malaysia, (2) southern coast of East Africa between Mozambique and southern Tanzania (SCEA), (3) inland Tanzania (IT), and (4) coastal region between Mombasa–Dar es Salaam and India (MDES). Baobabs from Tete included individuals with similarity to the SA cluster and others similar to MDES, and those from Senegal, the Mascarenes and Chennai equal likelihood of belonging to all four clusters (figure 3*a*,*b*).

At *K*=7, the clusters were: (1) SA, (2) SCEA, (3) inland Tanzania excluding Kilimanjaro (ITXK), (4) Kilimanjaro (KM), (5) Senegal, Chennai, Mascarenes and Malaysia (WA), (6) western India (WI), and (7) central India (CI). Individuals from the coastal region between Mombasa and Dar es Salaam were unable to be assigned to clusters, but showed similarities to clusters 4, 5, 6 and 7 (figure 4*a*,*b*).

The AMOVA supported the clustering patterns identified in the Structure analysis at *K*=2, 4 and 7 (table 2). For all tested population structures (from different values of *K*), genetic clustering was significantly higher than expected by chance. The partitioning of genetic variance among clusters, among populations within clusters, and within populations was similar across the different population structures that we tested. For subsequent analyses, we defined regions based on the structuring at *K*=7, as a more fine-scaled explanation of the population structure.

**Table 2.** *F* statistics, *p*-values, and proportions of variance at the level of individuals, populations and regions for AMOVA with populations clustered into regions based on Structure analysis at *K*=2, 4 and 7.

	*K*=2	*K*=4	*K*=7
variance among regions (%)	5.86	10.34	13.79
variance among populations (%)	11.42	6.49	2.52
variance within populations (%)	82.71	83.17	83.69
*F*_CT_	0.059 (*p*=0.00000)	0.103 (*p*=0.00000)	0.138 (*p*=0.00000)
*F*_SC_	0.121 (*p*=0.00000)	0.072 (*p*=0.00000)	0.029 (*p*=0.02931)
*F*_ST_	0.173 (*p*=0.00000)	0.168 (*p*=0.00000)	0.163 (*p*=0.00000)

Mantel tests and partial Mantel tests provided additional support for the clustering by the Structure analysis. The Mantel test for IBD across the entire range was significant (*R*_xy_=0.129; *p*=0.001), demonstrating that individuals from distant populations were more genetically divergent than individuals from nearby populations. When permutations were carried out only within *K*=7 genetic clusters, the Mantel test was no longer significant (*p*=0.142). The partial Mantel test of the *K*=7 genetic clusters, with geographical distance as a covariate, was significant (*R*_xy_=0.070; *p*=0.001). The latter tests showed that the pattern identified in the first Mantel test could be explained better as genetically cohesive clusters of populations, rather than a more gradual pattern of isolation by distance. Statistical support from AMOVA and Mantel tests aligned well with the Structure analysis, justifying use of the *K*=7 clusters for further analyses.

**Figure 5.**
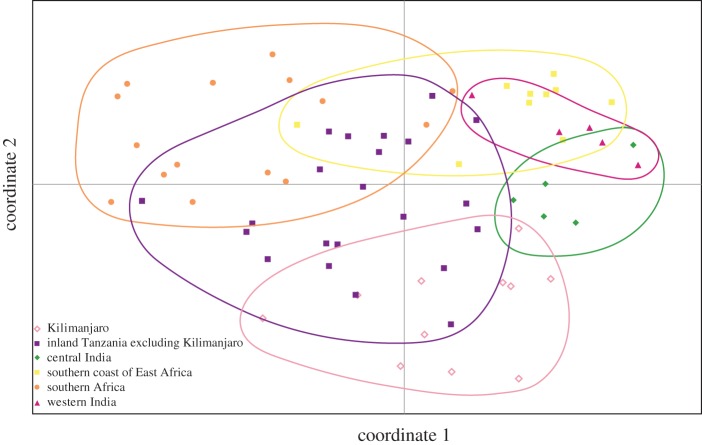
Graphical representation of the first and second axes (explaining 23.51% and 20.07% of variation, respectively) of a PCoA, based on genetic distances between all samples of *Adansonia digitata* for which there were no missing data.

The PCoA demonstrated weak differentiation between the clusters identified in the Structure analysis, with substantial overlap between clusters in coordinate space (figure 5). For the first principal coordinate (*x*-axis; explaining 23.51% of variation), individuals from the MDES, SCEA, KM, CI and WI clusters tended to have positive values; those from the SA cluster tended to have negative values, and those from ITXK were closer to zero. The second principal coordinate (*y*-axis; explaining 20.07% of variation), differentiated CI from WI individuals, and KM from SCEA, but with some overlap between adjacent clusters. Considering both coordinates, the WI individuals appear closer to those from SCEA and the CI individuals closer to MDES.

**Figure 6.**
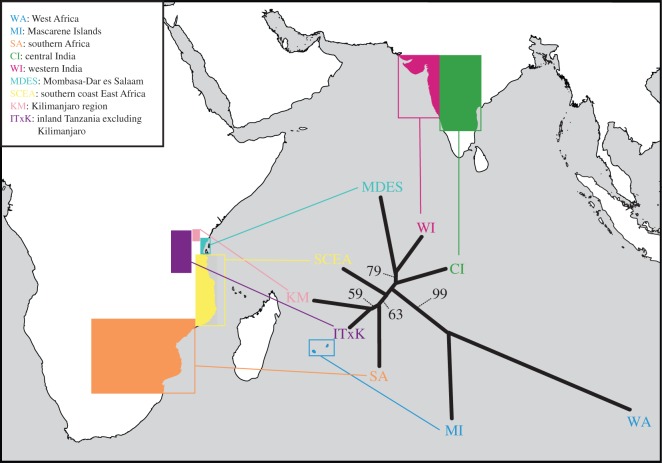
Unrooted neighbour-joining phylogeny of *Adansonia digitata* populations, based on genetic distances (*D*_C_) at the regional level. Geographical regions are identified by coloured boxes on the surrounding map. Bootstrap values above 50% are represented on branches.

The unrooted neighbour-joining tree of baobab individuals from eastern Africa, India, West Africa and Mascarene Islands supports inferences from Structure analysis (figure 6). The Indian and eastern African individuals clustered together, and the West African and Mascarene individuals clustered with relatively long branches leading to the latter two populations. Within the southern and eastern African regional populations, KM and ITXK formed a clade, as did SA and SCEA. The MDES cluster formed a clade with WI, and the CI cluster was sister to MDES and WI. The branches leading to the Indian populations were long relative to internal branches, consistent with the private alleles detected.

## 4. Discussion

### 4.1 Population structure and introductory relationships

The low genetic diversity in the Indian baobab populations compared with those from Africa is consistent with introduction to the Indian subcontinent. The Structure analysis showed clusters associating introduced populations in India with source populations in Africa at *K*=2 and 4. It also provides evidence for at least two introductions to India. The Chennai population appears most closely related to West African populations and might thus represent a modern introduction. The two major clusters from the Indian subcontinent (CI and WI) are closely related to (and likely derived from) the Mombasa-Dar es Salaam populations. However, they also differed from African clusters and contained private alleles.

There could be two reasons for the presence of private alleles. First, mutations may have occurred *in situ*, suggesting that these populations have been present for more than one generation. Hence the presence of private alleles could imply that the existing trees are not founding individuals. Around 20% of the trees sampled in India for the current study are above 1400 cm girth and, using Swart's estimate of baobab age based on girth measurement and radiocarbon dating [68], could be more than a thousand years old (electronic supplementary material, appendix S4). Second, the absence of evidence of a recent bottleneck could suggest introductions from multiple source populations, or earlier bottlenecks that could not be detected as deviations from mutation-drift equilibrium. These earlier bottlenecks could be the result of introduction by ancient humans.

Within the African clusters, the Structure analysis revealed individuals with ancestry from multiple clusters at *K*=4 for SCEA and ambiguously assigned individuals at *K*=7 for MDES, which either indicates dispersal from different regional clusters or the presence of unique individuals not matching other sampled populations. The phylogenetic analysis shows that MDES populations were the most likely African source for introductions to the Indian subcontinent, but the long branches leading to CI and WI may indicate relationships to other non-sampled sources, perhaps to those from further north near the Horn of Africa. However, the evidence of individuals assigned to both MDES and SCEA clusters suggests that both may have been source populations for the Indian introductions. The PCoA shows some clustering of populations, but with substantial overlap. This is consistent with substantial and repeated dispersals between African sources and Indian populations, preventing complete differentiation. The PCoA supports the close genetic similarity of WI and CI baobabs with the MDES cluster.

### 4.2 Correlations with historical trade routes and networks

The long history of trade networks across the Indian Ocean can provide evidence for reconstructing the introduction of baobabs from Africa to the Indian subcontinent and other regions of the Indian Ocean [37–39,69,70]. The PCoA shows close similarity between individuals from MDES and WI and CI regions. However, the Structure analysis provides evidence for SCEA as an additional source for introductions to India. The history of trade interactions between East African coastal centres and those in the Indian subcontinent suggests that the MDES populations were possibly the main source for introductions to CI, and the SCEA populations a significant source for WI introductions [46,52,71,72].

As noted earlier, the presence of private alleles in the Indian populations may indicate introductions from biogeographic regions of Africa not sampled in this study. These could be from the Sudanian Region or the Sahelian Transition Zone as per White's [69] chorological and vegetation mapping, or the Somalian and Ethiopian biogeographic regions as identified by Linder *et al.* [73]. The Somalian Region defined by Linder *et al.* extends across most of northeast Africa including the Horn, most of Kenya and the eastern half of Ethiopia; their Ethiopian Region includes significant elements of White's Sudanian and Afromontane (e.g. KM) regions. Hence the private alleles found in the Indian clusters could indicate introductions from populations in present-day Sudan, Ethiopia and Somalia, all of which have had histories of interactions with the Indian subcontinent extending over more than four millennia [74].

The presence of individuals in Tete-Mocuba with ancestry in the SA and SCEA clusters suggests that baobab dispersal occurred from these neighbouring populations into this area (figures 3 and 4; *K*=4, 7). These source cluster areas match White's [69] Zambezian Region, Kalahari-Highveld Transition Zone and Zanzibar-Inhambane Mosaic. Following Linder *et al*.'s [73] biogeographic classification, these source clusters occur in the Zambezian Region incorporating Mozambique; the Southern African Region incorporating the Kalahari-Namib and the South African Zones; and the Kilimanjaro Zone. The presence of individuals of divergent ancestries in this population corresponds with the history of long-term trade networks that connected southern and eastern inland regions with the East African coast [46,74]. A striking corroboration of genetic and historic evidence of the inland and coastal interactions is the presence of different cluster ancestries among the individuals from Tete-Mocuba in inland Mozambique. Tete, which is located on the banks of the Zambezi River, was the farthest inland trading centre that could be reached by boat from the coast; beyond this point, the Zambezi rapids made it difficult for boat navigation. Tete was an important trading centre for African people that were captured and sold as slaves to Portuguese and Swahili traders [75]. Captives were brought to Tete from places around Lake Malawi, present-day eastern Zimbabwe and the southern highlands of Tanzania, and transported by boat to trading ports such as Quelimane, Cabo Delgado, Ilha do Moçambique, Kilwa and Zanzibar [70].

The assignment of individuals from West Africa, Mascarene Islands, southeast India and Malaysia to the same cluster in the Structure analysis at *K*=7 (figure 4), and to the same clade in the intraspecific phylogenetic tree (figure 6), suggests that West Africa may be the source of introductions to these places. However, this inference needs to be treated with caution since it is based on limited samples. The history of trade between West Africa and other parts of the Indian Ocean also extends over several millennia through a combination of land and oceanic routes and networks across the Sahel to ports on the Mediterranean Sea and Red Sea [76]. From the sixteenth century onwards, European colonial networks linked the Atlantic and Indian Oceans via the Cape of Good Hope [72,76,77].

### 4.3 Inferences on timing of baobab introductions

The presence of private alleles in the Indian clusters may represent introductions further back in time or indicate introductions from baobab populations in northeast Africa. Transoceanic drift could have been a factor. Baobab fruit could have floated on ocean currents to western Indian shores and spread inland without human assistance. This would have been a slow process resulting in strong genetic divergence, or even speciation. However, the lack of species-level divergence between African and Indian baobabs makes this scenario less probable. It is more likely that the ancient introductions were from northeast Africa. Baobabs occur in Sudanian–Sahelian transition zone which includes present-day Sudan, Ethiopia and Somalia. These are the areas where food crops such as sorghum, pearl and finger millets, cowpea, hyacinth bean and useful trees such as the tamarind originated [32,35,37,46], and arrived in the Indian subcontinent at least 4000 years ago [39–41,44,72]. It is likely that ancient Africans belonging to farming, nomadic and fishing communities of this zone were familiar with the numerous uses of baobabs for food, medicinal, artisanal and symbolic purposes [20,21,23,78]. Given the similar time scales, a more parsimonious explanation for the introduction of baobabs to India is that these people carried the lightweight, nutritious and hardy baobab fruit with them as they travelled overland or on sea journeys across the Indian Ocean.

**Figure 7.**
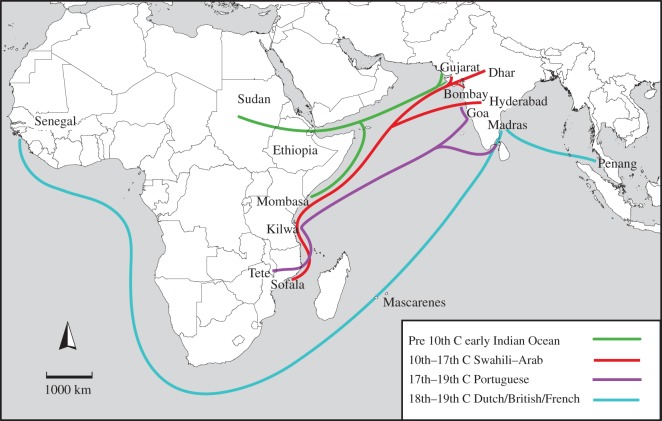
Inferred pathways of introduction of baobabs from continental Africa to India and beyond in relation to oceanic trade during different historic periods.

Figure 7 shows the pathways of introduction of baobabs to India and beyond in relation to oceanic trade during different historical periods. Ancient and early historical introductions are consistent with trade connections between Sudan and Ethiopia in northeast Africa and western India. The expansion of Swahili–Arab trade and cultural networks around the western Indian Ocean between the tenth and seventeenth centuries matches with evidence of MDES and SCEA sources in the Indian clusters [77]. The close position of MDES and CI baobab clusters in the population-level phylogeny may reflect ancestry of the CI populations in the Sudanian–Sahelian transition zone. This corresponds with historical evidence of regular recruitment of soldiers from Ethiopia and Sudan during this period for the armies of Muslim kingdoms in central India [31].

The activities of the Portuguese in the Indian Ocean region between the sixteenth and nineteenth centuries provide insights for the short genetic distance between SCEA and WI clusters. During this period, Portuguese colonists and traders in eastern Africa obtained soldiers and slaves from present-day Malawi, northern Mozambique and southern Tanzania for their territorial enclaves in western India [71,79]. As the enslaved people were marched from these areas to the ports, they would have picked up the fallen baobab fruit and eaten them along the way. Being familiar with the tree, they would have known the nutritive qualities of its mature fruit, its lightness and durability of the pod, and the long-term viability and edibility of the pulp and seeds, and thus carried the fruit with them on their journey to the seaports and beyond [80]. The presence of individuals from multiple clusters in the southeast African coastal populations is most strongly reflective of the Portuguese slave trade in East Africa during this period, when men and women captured from places around Lake Malawi and inland Mozambique were brought to Tete and then moved to ports such as Quelimane and Ilha do Moçambique, to be shipped to colonial sugarcane plantations in the Mascarene Islands and Brazil [79]. The ambiguous cluster assignments in the MDES cluster may be the result of baobab introductions from various sampled and unsampled populations. This would be consistent with heavy traffic related to slave trading by Swahili–Arab traders during this period. These traders obtained captives from highland and inland regions of southern Tanzania and shipped them from Kilwa, Pemba and Zanzibar to sugarcane plantations in Réunion and Mauritius, and date palm (*Phoenix sylvestris*) plantations in Oman and around the Persian Gulf [70].

The grouping of baobab individuals from West Africa, Mascarene Islands, southeast India and Malaysia in the same genetic clusters is consistent with the history of Dutch, English and French colonial networks between Africa and Asia. During the eighteenth and nineteenth centuries, both the English and Dutch East India Companies recruited ship crews, soldiers and labourers from West Africa for their factories and forts in southeast India, Ceylon and the East Indies [31,81]. In addition, English, Dutch and French botanists travelled between their colonies in West Africa, the Mascarenes, South and Southeast Asia, often transferring plants for commercial production or ornamental purposes [40]. A combination of both kinds of human agency may have introduced West African baobabs in the Mascarenes (Mauritius), eastern India (Madras) and Southeast Asia (Penang).

## 5. Conclusion

By combining genetic analysis of baobabs with historical evidence of oceanic trade, this study provides three important findings that improve current understanding of the history of their dispersal across the Indian Ocean.

First, it shows that there have been multiple instances of dispersal from continental Africa to the Indian subcontinent, possibly even extending back into prehistoric times. Second, we infer that the dispersal of baobabs in the Indian subcontinent and around the Indian Ocean was due to the agency of African migrants. Their long-standing familiarity with the baobab fruit and the tree's ubiquitous presence along inland trade routes would have made it a free and important food for surviving long journeys on land and by sea. The evidence of multiple introductions, and sources from different regions of continental Africa show, without doubt, that the presence of baobabs in the Indian subcontinent, the Mascarenes and Penang signals the forgotten history of African migrants to these places [82].

Finally, our study highlights the need for further genetic analyses of African baobab populations incorporating additional samples from West Africa, Somalia, Ethiopia, Sudan, coastal Yemen, Oman, Iran, Pakistan and Sri Lanka. These analyses can be combined with more detailed historical, cultural and linguistic evidence to establish new hypotheses of baobab dispersal across the Indian Ocean.

## Supplementary Material

Appendix S1 Collection localities of Adansonia digitata individuals used in this study and genetic cluster assignments based on STRUCTURE analysis. Appendix S2 Details of Adansonia digitata populations used in this study and predominant genetic cluster assignments of individuals within poopulations based on STRUCTURE analysis. Appendix S3 Values of Gis for populations of Adansonia digitata for each microsatellite locus, and across all loci.

## Supplementary Material

Appendix S4 Collection data and size of baobab trees sampled in India, reported as height, diameter, and girth at breast height (GBH).

## Supplementary Material

Appendix S5 Microsatellite data (reported as size of PCR product including fluorochrome) for each individual.

## Data accessibility

The datasets supporting this article have been uploaded as the electronic supplementary material.
