# Supplementary material for: The history of introduction of the African baobab (Adansonia digitata, Malvaceae: Bombacoideae) in the Indian subcontinent
Source: R Soc Open Sci. 2015 Sep 9;2(9):150370. doi: 10.1098/rsos.150370 (PMC4593694; doi:10.1098/rsos.150370)
Supplement: Appendix S1 Collection localities of Adansonia digitata individuals used in this study and genetic cluster assignments based on STRUCTURE analysis. Appendix S2 Details of Adansonia digitata populations used in this study and predominant genetic cluster assignments of individuals within poopulations  [file rsos150370supp1.doc]

Appendix S1: Collection details and vouchers of samples used in this study and genetic cluster assignments based on Structure analysis. Cluster assignment is considered ambiguous if the Q-value is less than 0.75.

| Population | Collector | Collection Number | Voucher details | Species | Latitude | Longitude | Cluster (*K*=2) | Cluster (*K*=4) | Cluster (*K*=7) |
| --- | --- | --- | --- | --- | --- | --- | --- | --- | --- |
| Pretoria Botanic Gardens, South Africa | J. Pettigrew | 402 | PRE 0857831-0 | *kilima* | 26.18°S | 28.0091°E | 2 | 4 | 1 |
| Kruger NP, South Africa | J. Pettigrew | 374 |  | *digitata* | 22.6058°S | 29.92713°E | 2 | 4 | 1 |
|  | J. Pettigrew | 375 |  | *digitata* | 22.5982°S | 29.92899°E | 2 | 4 | 1 |
|  | J. Pettigrew | 376 |  | *digitata* | 22.4967°S | 29.97986°E | 2 | 4 | 1 |
|  | J. Pettigrew | 377 |  | *kilima* | 22.4729°S | 29.99045°E | 2 | ambiguous | ambiguous |
|  | J. Pettigrew | 379 |  | *digitata* | 22.3414°S | 30.04201°E | 2 | ambiguous | ambiguous |
|  | J. Pettigrew | 380 |  | *kilima* | 22.3752°S | 30.0639°E | 2 | 4 | 1 |
|  | J. Pettigrew | 383 |  | *kilima* | 22.6043°S | 30.17218°E | 2 | 4 | 1 |
|  | J. Pettigrew | 387 |  | *kilima* | 22.7212°S | 31.03397°E | 2 | 4 | 1 |
|  | J. Pettigrew | 390 |  | *digitata* | 22.4923°S | 31.20596°E | 2 | 4 | 1 |
| Beira, Mozambique | C. Kull & H. Rangan | 15 |  | *digitata/kilima* | 19.4575°S | 34.38722°E | 2 | 4 | 1 |
|  | C. Kull & H. Rangan | 20 |  | *digitata/kilima* | 20.1086°S | 34.75861°E | 2 | 4 | 1 |
| Caprivi Strip, Namibia | J. Pettigrew | 444 |  | *digitata* | 18.1106°S | 21.559°E | 2 | 4 | 1 |
|  | J. Pettigrew | 458 |  | *digitata* | 17.8997°S | 25.85572°E | 2 | 4 | 1 |
|  | J. Pettigrew | 488 | WIS v0263271 | *kilima* | 17.6177°S | 24.3991°E | ambiguous | 4 | ambiguous |
| Tete, Mozambique | C. Kull & H. Rangan | 23 | MEL 2370401A | *digitata/kilima* | 16.8769°S | 33.27194°E | 2 | 4 | ambiguous |
|  | C. Kull & H. Rangan | 24 |  | *digitata/kilima* | 16.5534°S | 33.94395°E | 2 | 4 | 1 |
|  | C. Kull & H. Rangan | 25 |  | *digitata/kilima* | 16.5549°S | 33.94498°E | 1 | 2 | 3 |
|  | C. Kull & H. Rangan | 26 |  | *digitata/kilima* | 16.1672°S | 33.64222°E | ambiguous | ambiguous | ambiguous |
|  | C. Kull & H. Rangan | 27 | MEL 2370333A | *digitata/kilima* | 16.2705°S | 33.5453°E | 2 | 4 | 1 |
| Mocuba, Mozambique | C. Kull & H. Rangan | 28 |  | *digitata/kilima* | 16.7717°S | 37.03111°E | ambiguous | ambiguous | ambiguous |
| Nampula to Pemba, Mozambique | C. Kull & H. Rangan | 29 | MEL 2370364A | *digitata/kilima* | 15.1336°S | 39.3925°E | 1 | 2 | 3 |
|  | C. Kull & H. Rangan | 30 |  | *digitata/kilima* | 15.1183°S | 39.40944°E | 2 | 4 | 1 |
|  | C. Kull & H. Rangan | 31 |  | *digitata/kilima* | 14.9747°S | 40.45833°E | 1 | 2 | 3 |
|  | C. Kull & H. Rangan | 32 | MEL 2370395A | *digitata/kilima* | 15.0335°S | 40.68475°E | 1 | 2 | 3 |
|  | C. Kull & H. Rangan | 34 |  | *digitata/kilima* | 15.0408°S | 40.7281°E | 1 | 2 | 3 |
|  | C. Kull & H. Rangan | 35 | MEL 2370366A | *digitata/kilima* | 14.7472°S | 39.95652°E | 1 | 2 | 3 |
|  | C. Kull & H. Rangan | 36 |  | *digitata/kilima* | 13.8414°S | 39.88194°E | 1 | 2 | 3 |
|  | C. Kull & H. Rangan | 37 | MEL 2370351A | *digitata/kilima* | 13.0994°S | 40.46768°E | 1 | 2 | 3 |
|  | C. Kull & H. Rangan | 38 | MEL 2357521A | *digitata/kilima* | 12.9696°S | 40.55193°E | 1 | 2 | 3 |
|  | C. Kull & H. Rangan | 39 |  | *digitata/kilima* | 12.9717°S | 40.54694°E | 1 | 2 | 3 |
|  | C. Kull & H. Rangan | 40 | MEL 2357526A | *digitata/kilima* | 12.9712°S | 40.53811°E | 1 | 2 | 3 |
|  | C. Kull & H. Rangan | 41 |  | *digitata/kilima* | 12.9631°S | 40.52596°E | 1 | 6 | 3 |
|  | C. Kull & H. Rangan | 42 |  | *digitata/kilima* | 12.9614°S | 40.52167°E | 1 | 2 | 3 |
|  | C. Kull & H. Rangan | 43 |  | *digitata/kilima* | 13.0547°S | 40.52778°E | 1 | 2 | 3 |
|  | C. Kull & H. Rangan | 44 |  | *digitata/kilima* | 12.9889°S | 40.52944°E | 1 | 2 | 3 |
| Kilwa to Mtwara, Tanzania | C. Kull & H. Rangan | 67 |  | *digitata/kilima* | 8.9326°S | 39.52623°E | ambiguous | 2 | 3 |
|  | C. Kull & H. Rangan | 68 | MEL 2370363A | *digitata/kilima* | 8.92717°S | 39.5204°E | 1 | ambiguous | ambiguous |
|  | C. Kull & H. Rangan | 69 |  | *digitata/kilima* | 8.95951°S | 39.49618°E | 1 | 6 | 3 |
|  | C. Kull & H. Rangan | 70 | MEL 2357520A | *digitata/kilima* | 8.96167°S | 39.49944°E | 1 | 6 | 3 |
|  | C. Kull & H. Rangan | 71 |  | *digitata/kilima* | 8.96274°S | 39.50341°E | 1 | ambiguous | ambiguous |
|  | C. Kull & H. Rangan | 72 |  | *digitata/kilima* | 8.87583°S | 39.4934°E | 1 | ambiguous | ambiguous |
|  | C. Kull & H. Rangan | 73 |  | *digitata/kilima* | 9.52056°S | 39.47556°E | 1 | 2 | 3 |
|  | C. Kull & H. Rangan | 74 |  | *digitata/kilima* | 9.63°S | 39.5014°E | 1 | 2 | ambiguous |
|  | C. Kull & H. Rangan | 75 |  | *digitata/kilima* | 9.97028°S | 39.70417°E | 1 | 2 | 3 |
|  | C. Kull & H. Rangan | 76 |  | *digitata/kilima* | 10.2383°S | 39.95528°E | 1 | 2 | 3 |
|  | C. Kull & H. Rangan | 77 |  | *digitata/kilima* | 10.2814 °S | 40.17984°E | 1 | 2 | 3 |
|  | C. Kull & H. Rangan | 78 |  | *digitata/kilima* | 10.3442°S | 40.2475°E | 1 | 2 | 3 |
|  | C. Kull & H. Rangan | 79 | MEL 2370343A | *digitata/kilima* | 10.3611°S | 40.41278°E | 1 | 2 | 3 |
| Morogoro, Tanzania | J. Pettigrew | 327 |  | *digitata* | 6.58034°S | 38.33289°E | 1 | ambiguous | ambiguous |
|  | C. Kull & H. Rangan | 64 |  | *digitata/kilima* | 6.95806°S | 37.29944°E | 1 | 2 | 3 |
|  | C. Kull & H. Rangan | 65 |  | *digitata/kilima* | 7.28444°S | 37.84528°E | 1 | 2 | 3 |
|  | C. Kull & H. Rangan | 66 | MEL 2370365A | *digitata/kilima* | 7.86556°S | 38.50083°E | 1 | ambiguous | ambiguous |
| Ruaha River, Tanzania | C. Kull & H. Rangan | 62 |  | *digitata/kilima* | 7.56167°S | 36.68472°E | 2 | 1 | 6 |
|  | C. Kull & H. Rangan | 63 | MEL 2370334A, MEL 2370365A (Carpological) | *digitata/kilima* | 7.52722°S | 36.65611°E | 1 | 2 | 3 |
|  | J. Pettigrew | 331 |  | *kilima* | 7.56943°S | 36.71343°E | 2 | ambiguous | ambiguous |
|  | J. Pettigrew | 343 |  | *kilima* | 7.3319°S | 35.74078°E | 2 | ambiguous | ambiguous |
|  | J. Pettigrew | 350 |  | *kilima* | 7.16303°S | 35.76263°E | 2 | 1 | 6 |
|  | J. Pettigrew | 355 |  | *kilima* | 6.83112°S | 36.05655°E | 2 | 1 | 6 |
| Dodoma to Kondoa, Tanzania | J. Pettigrew | 356 |  | *kilima* | 5.93018°S | 35.74407°E | ambiguous | ambiguous | ambiguous |
|  | J. Pettigrew | 357 |  | *kilima* | 5.8031°S | 35.80121°E | 2 | 1 | 6 |
|  | J. Pettigrew | 358 |  | *kilima* | 5.67787°S | 35.82927°E | 2 | 1 | ambiguous |
|  | J. Pettigrew | 359 |  | *kilima* | 5.19951°S | 35.87882°E | 2 | 1 | 6 |
|  | J. Pettigrew | 360 |  | *kilima* | 5.14516°S | 35.83793°E | ambiguous | 1 | 6 |
|  | J. Pettigrew | 361 |  | *digitata* | 5.10347°S | 35.82084°E | 2 | 1 | 6 |
|  | J. Pettigrew | 362 |  | *digitata/kilima* | 4.98298°S | 35.7982°E | 2 | 1 | 6 |
|  | J. Pettigrew | 363 |  | *kilima* | 4.98298°S | 35.7982°E | 2 | 1 | 6 |
|  | C. Kull & H. Rangan | 50 | MEL 2370380A, MEL 2370575A (Carpological) | *digitata/kilima* | 4.86511°S | 35.82814°E | 2 | 1 | 6 |
|  | C. Kull & H. Rangan | 51 |  | *digitata/kilima* | 4.86742°S | 35.82737°E | 2 | ambiguous | 6 |
|  | C. Kull & H. Rangan | 52 |  | *digitata/kilima* | 4.86802°S | 35.82738°E | 2 | 1 | 6 |
|  | C. Kull & H. Rangan | 53 | MEL 2370340A, MEL 2370574A (Carpological) | *digitata/kilima* | 4.71917°S | 35.89722°E | 2 | 1 | ambiguous |
|  | C. Kull & H. Rangan | 54 |  | *digitata/kilima* | 4.70111°S | 35.87722°E | 2 | 1 | 6 |
|  | C. Kull & H. Rangan | 55 |  | *digitata/kilima* | 4.70028°S | 35.87861°E | 2 | 1 | 6 |
|  | C. Kull & H. Rangan | 56 |  | *digitata/kilima* | 4.69917°S | 35.87917°E | 2 | 1 | 6 |
|  | C. Kull & H. Rangan | 57 |  | *digitata/kilima* | 5.17389°S | 35.86306°E | 2 | 4 | 6 |
|  | C. Kull & H. Rangan | 58 |  | *digitata/kilima* | 5.58917°S | 35.83833°E | 2 | 1 | 6 |
|  | C. Kull & H. Rangan | 59 |  | *digitata/kilima* | 6.12972°S | 36.205°E | 2 | 1 | ambiguous |
|  | C. Kull & H. Rangan | 60 |  | *digitata/kilima* | 6.12972°S | 36.205°E | 2 | 4 | ambiguous |
| Tarangire, Tanzania | J. Pettigrew | 368 |  | *digitata* | 4.09309°S | 35.76496°E | 2 | 1 | 6 |
|  | J. Pettigrew | 369 |  | *kilima* | 3.76588°S | 35.87265°E | 2 | 1 | 6 |
|  | J. Pettigrew | 370 |  | *kilima* | 3.7659°S | 35.87265°E | 2 | 1 | 6 |
|  | J. Pettigrew | 371 |  | *kilima* | 3.77548°S | 35.96775°E | 2 | ambiguous | ambiguous |
|  | J. Pettigrew | 372 |  | *kilima* | 3.76786°S | 35.9693°E | 2 | 1 | 6 |
|  | C. Kull & H. Rangan | 46 |  | *digitata/kilima* | 3.67222°S | 35.94861°E | 2 | ambiguous | ambiguous |
|  | C. Kull & H. Rangan | 47 |  | *digitata/kilima* | 3.98639°S | 35.78833°E | 2 | 1 | ambiguous |
|  | C. Kull & H. Rangan | 48 |  | *digitata/kilima* | 4.22111°S | 35.74861°E | 2 | 1 | 6 |
|  | C. Kull & H. Rangan | 49 |  | *digitata/kilima* | 4.35667°S | 35.74889°E | 2 | 1 | 6 |
| Mombasa to Dar es Salaam, Kenya & Tanzania | C. Kull & H. Rangan | 45 |  | *digitata/kilima* | 6.77222°S | 39.21306°E | 1 | 3 | ambiguous |
|  | J. Pettigrew | 292 |  | *digitata/kilima* | 4.65816°S | 39.36837°E | ambiguous | ambiguous | ambiguous |
|  | J. Pettigrew | 293 |  | *digitata* | 3.33222°S | 40.0125°E | 1 | 3 | ambiguous |
|  | J. Pettigrew | 294 |  | *digitata* | 3.32267°S | 40.0241°E | 1 | 3 | ambiguous |
|  | J. Pettigrew | 295 |  | *digitata* | 3.67142°S | 39.82312°E | 1 | 3 | ambiguous |
| Kilimanjaro, Kenya & Tanzania | J. Pettigrew | 296 |  | *digitata/kilima* | 3.42218°S | 38.60326°E | 2 | 1 | 4 |
|  | J. Pettigrew | 304 |  | *kilima* | 3.39113°S | 37.74753°E | 2 | 1 | ambiguous |
|  | J. Pettigrew | 308 |  | *kilima* | 2.21734°S | 37.73838°E | 2 | 1 | 4 |
|  | J. Pettigrew | 310 |  | *kilima* | 2.28368°S | 37.82301°E | 2 | 1 | 4 |
|  | J. Pettigrew | 313 |  | *kilima* | 2.33742°S | 37.88357°E | ambiguous | ambiguous | 4 |
|  | J. Pettigrew | 314 |  | *kilima* | 2.37772°S | 37.90034°E | 2 | 1 | ambiguous |
|  | J. Pettigrew | 317 |  | *kilima* | 3.38028°S | 37.43264°E | 2 | ambiguous | ambiguous |
|  | J. Pettigrew | 318 |  | *kilima* | 3.38063°S | 37.43269°E | 2 | 1 | 4 |
|  | J. Pettigrew | 319 |  | *kilima* | 3.35199°S | 37.34459°E | 2 | 1 | 4 |
|  | J. Pettigrew | 322 |  | *kilima* | 3.4527°S | 37.53154°E | 2 | 1 | 4 |
|  | J. Pettigrew | 323 |  | *kilima* | 3.4527°S | 37.53154°E | 2 | 1 | 4 |
|  | J. Pettigrew | 325 |  | *kilima* | 4.08996°S | 37.75761°E | 2 | 1 | 4 |
|  | J. Pettigrew | 326 |  | *kilima* | 4.79078°S | 38.19673°E | ambiguous | ambiguous | ambiguous |
| Senegal | P. Danthu | Adi2 | MEL 2357519A | *digitata* | 14°N | 14°W | 2 | ambiguous | 2 |
| Indian Ocean Islands | H. Rangan & C. Kull | MRU001 | MEL 2370388A (Carpological) | *digitata/kilima* | 20.1°S | 57.56667°E | 2 | ambiguous | 2 |
|  | P. Danthu | MRU002 |  | *digitata/kilima* | 20.2833°S | 57.4°E | 1 | ambiguous | ambiguous |
|  | P. Danthu | RUNB002 |  | *digitata/kilima* | 21°S | 55°E | 1 | ambiguous | ambiguous |
| Mumbai to Goa, India | H. Rangan | IND0411 | MEL 2370370A, MEL 2370506A (Carpological) | *digitata/kilima* | 14.97783°N | 75.33322°E | 1 | 3 | 7 |
|  | H. Rangan | IND0412 | MEL 2370369A | *digitata/kilima* | 14.97783°N | 75.33322°E | 1 | ambiguous | ambiguous |
|  | H. Rangan | IND0413 |  | *digitata/kilima* | 14.97783°N | 75.33322°E | 1 | 3 | 7 |
|  | H. Rangan | IND0415 |  | *digitata/kilima* | 18.89875°N | 72.81321°E | 1 | 3 | 7 |
| Gujarat, India | H. Rangan | IND0421 |  | *digitata/kilima* | 23.11478°N | 69.54575°E | 1 | 3 | 7 |
|  | H. Rangan | IND0422 |  | *digitata/kilima* | 22.86588°N | 69.35667°E | 1 | ambiguous | ambiguous |
|  | H. Rangan | IND0423 | MEL 2370389A | *digitata/kilima* | 22.45159°N | 69.09851°E | ambiguous | ambiguous | ambiguous |
|  | H. Rangan | IND0424 |  | *digitata/kilima* | 21.50708°N | 70.13832°E | 1 | 3 | 7 |
|  | H. Rangan | IND0425 | MEL 2370390A, MEL 2370501A (Carpological) | *digitata/kilima* | 21.50708°N | 70.13832°E | 1 | 3 | 7 |
|  | H. Rangan | IND0426 | MEL 2370386A | *digitata/kilima* | 21.50708°N | 70.13832°E | 1 | 3 | 7 |
|  | H. Rangan | IND0427 | MEL 2370323A | *digitata/kilima* | 21.11878°N | 70.11668°E | 1 | 3 | 7 |
|  | H. Rangan | IND0428 | MEL 2370326A | *digitata/kilima* | 21.10132°N | 70.16943°E | ambiguous | ambiguous | ambiguous |
|  | H. Rangan | IND0429 | MEL 2370387A | *digitata/kilima* | 21.06623°N | 70.53287°E | 1 | 3 | 7 |
|  | H. Rangan | IND0430 |  | *digitata/kilima* | 20.90652°N | 70.53568°E | 1 | 3 | 7 |
|  | H. Rangan | IND0431 | MEL 2370344A | *digitata/kilima* | 20.70557°N | 70.90715°E | 1 | 3 | 7 |
|  | H. Rangan | IND0432 | MEL 2370399A | *digitata/kilima* | 20.74135°N | 71.07103°E | 1 | 3 | 7 |
|  | H. Rangan | IND0433 | MEL 2370353A | *digitata/kilima* | 20.86323°N | 71.36642°E | 1 | 3 | 7 |
|  | H. Rangan | IND0434 | MEL 2370345A | *digitata/kilima* | 20.899°N | 71.39068°E | 1 | 3 | 7 |
|  | H. Rangan | IND0435 | MEL 2370372A | *digitata/kilima* | 21.68213°N | 72.27983°E | 1 | 3 | 7 |
|  | H. Rangan | IND0436 | MEL 2370330A | *digitata/kilima* | 21.74665°N | 72.14073°E | 1 | 3 | 7 |
|  | H. Rangan | IND0437 | MEL 2370327A | *digitata/kilima* | 21.77193°N | 72.1421°E | 1 | 3 | 7 |
|  | H. Rangan | IND0438 | MEL 2370356A | *digitata/kilima* | 23.023°N | 72.56455°E | 1 | 3 | 7 |
|  | H. Rangan | IND0439 | MEL 2370350A | *digitata/kilima* | 23.02103°N | 72.57823°E | 1 | 3 | 7 |
| Hyderabad, India | H. Rangan | IND0450 | MEL 2370341A, MEL 2370510A (Carpological) | *digitata/kilima* | 17.42698°N | 78.6139°E | 1 | ambiguous | ambiguous |
|  | H. Rangan | IND0455 | MEL 2370391A | *digitata/kilima* | 17.39285°N | 78.41083°E | 1 | ambiguous | ambiguous |
| Nagpur, India | H. Rangan | IND0211 | MEL 2370381A | *digitata/kilima* | 21.15492°N | 79.05258°E | ambiguous | ambiguous | 12 |
|  | H. Rangan | IND3110 |  | *digitata/kilima* | 21.16778°N | 79.07389°E | 1 | ambiguous | ambiguous |
| Dhar District, India | H. Rangan | IND0440 | MEL 2370359A | *digitata/kilima* | 22.3281°N | 75.38197°E | ambiguous | 3 | 5 |
|  | H. Rangan | IND0441 | MEL 2370355A, MEL 2370441A (Carpological) | *digitata/kilima* | 22.32975°N | 75.39408°E | 1 | 3 | 5 |
|  | H. Rangan | IND0442 |  | *digitata/kilima* | 22.33062°N | 75.39408°E | ambiguous | 3 | 5 |
|  | H. Rangan | IND0443 | MEL 2370329A | *digitata/kilima* | 22.3486°N | 75.39385°E | 1 | ambiguous | ambiguous |
|  | H. Rangan | IND0444 | MEL 2370367A | *digitata/kilima* | 22.34643°N | 75.3968°E | 1 | 3 | 5 |
|  | H. Rangan | IND0445 | MEL 2370328A | *digitata/kilima* | 22.33777°N | 75.40103°E | ambiguous | 3 | 5 |
|  | H. Rangan | IND0446 | MEL 2370325A | *digitata/kilima* | 22.32238 °N | 75.40123 °E | 1 | 3 | 5 |
| Chennai, India | H. Rangan | IND0449 | MEL 2370398A | *digitata/kilima* | 13.08333°N | 80.26667°E | 2 | ambiguous | 2 |
| Penang, Malaysia | H. Rangan | MAL1 |  | *digitata/kilima* | 5.422114°N | 100.3123°E | 2 | 4 | 2 |

Appendix S2: Collection localities of *Adansonia digitata* populations used in this study and genetic cluster assignments based on Structure analysis. Cluster assignment is considered ambiguous if the highest Q-value is less than 0.75.

| Population | Number of samples | Mean Latitude | Mean Longitude | Predominant Cluster (*K* = 2) | Predominant Cluster (*K* = 4) | Predominant Cluster (*K* = 7) |
| --- | --- | --- | --- | --- | --- | --- |
| Pretoria Botanic Gardens, South Africa | 1 | 26.18°S | 28.0091°E | 2 | 4 | 1 |
| Kruger NP, South Africa | 9 | 22.5231°S | 30.2605°E | 2 | 4 | 1 |
| Beira, Mozambique | 2 | 19.7831°S | 34.5096°E | 2 | 4 | 1 |
| Caprivi Strip, Namibia | 3 | 17.876°S | 23.9379°E | 2 | 4 | 1 |
| Tete, Mozambique | 5 | 16.4846°S | 33.27194°E | 2 | 4 | 1 |
| Mocuba, Mozambique | 1 | 16.7717°S | 37.03111°E | ambiguous | ambiguous | ambiguous |
| Nampula to Pemba, Mozambique | 15 | 13.8580°S | 40.3147°E | 1 | 2 | 3 |
| Kilwa to Mtwara, Tanzania | 13 | 9.5358°S | 39.7474°E | 1 | 2 | 3 |
| Morogoro, Tanzania | 4 | 7.1721°S | 37.9946°E | 1 | 2 | 3 |
| Ruaha River, Tanzania | 6 | 7.3307°S | 36.2690°E | 2 | 1 | 6 |
| Dodoma to Kondoa, Tanzania | 19 | 5.2246°S | 35.8755°E | 2 | 1 | 6 |
| Tarangire, Tanzania | 9 | 3.9338°S | 35.8535°E | 2 | 1 | 6 |
| Mombasa to Dar es Salaam, Kenya & Tanzania | 5 | 4.3513°S | 39.6882°E | 1 | 3 | ambiguous |
| Kilimanjaro, Kenya & Tanzania | 13 | 3.2253°S | 37.7633°E | 2 | 1 | 4 |
| Senegal | 1 | 14°N | 14°W | 2 | ambiguous | 2 |
| Mascarene Islands | 3 | 20.4611°S | 56.6556°E | 1 | ambiguous | ambiguous |
| Mumbai to Dharwad, India | 4 | 15.9581°N | 74.7032°E | 1 | 3 | 7 |
| Gujarat, India | 19 | 22.8077°N | 70.8532°E | 1 | 3 | 7 |
| Hyderabad, India | 2 | 17.4099°N | 78.5124°E | 1 | ambiguous | ambiguous |
| Nagpur, India | 2 | 21.1614°N | 79.0632°E | 1 | ambiguous | 2 |
| Dhar District, India | 7 | 22.3348°N | 75.3947°E | 1 | 3 | 5 |
| Chennai, India | 1 | 13.08333°N | 80.26667°E | 2 | ambiguous | 2 |
| Penang, Malaysia | 1 | 5.4221°N | 100.3123°E | 2 | 4 | 2 |
